# Binding Does Not Mean Stabilizing: High-Affinity Porphyrins Exhibit Opposite Effects on G‑Quadruplex Stability

**DOI:** 10.1021/acs.jpclett.6c02493

**Published:** 2026-09-14

**Authors:** Cristina Carucci, Monica Mura, Francesca Cherchi, Giuseppe Satta, Aamir Saeed, Barbara Jachimska, Francesca Mocci, Massimo Carraro, Andrea Salis

**Affiliations:** † Department of Chemical and Geological Sciences, 3111 University of Cagliari & CSGI, Cittadella Universitaria, S.S. 554 bivio Sestu, 09042 Monserrato (Cagliari), Italy; ‡ Department of Chemical, Physical, Mathematical and Natural Sciences, University of Sassari, Via Vienna 2, Sassari 07100, Italy; § Jerzy Haber Institute of Catalysis and Surface Chemistry Polish Academy of Sciences, Krakow 30-239, Poland

## Abstract

Binding affinity is often used as a predictor of G-quadruplex (G4) stabilization. Here, we show that ligand binding and quadruplex stabilization can be thermodynamically decoupled. Two closely related tetracationic porphyrins bind the human telomeric G4 with comparable affinities approaching 10^6^ M^–1^ but induce opposite thermal responses. TMPyP4 stabilizes the quadruplex (+9 °C), whereas PL7 destabilizes it (−6 °C) despite a similar binding free energy. Thermodynamic analysis reveals distinct binding mechanisms, with TMPyP4 association driven primarily by favorable enthalpy and PL7 binding dominated by entropy. Temperature-dependent circular dichroism measurements indicate that PL7 promotes redistribution within the telomeric G4 conformational ensemble rather than stabilization of the native hybrid fold. These findings demonstrate that high affinity does not necessarily imply G4 stabilization and establish affinity and thermal stabilization as independent descriptors of ligand–quadruplex interactions.

## Graphical abstract

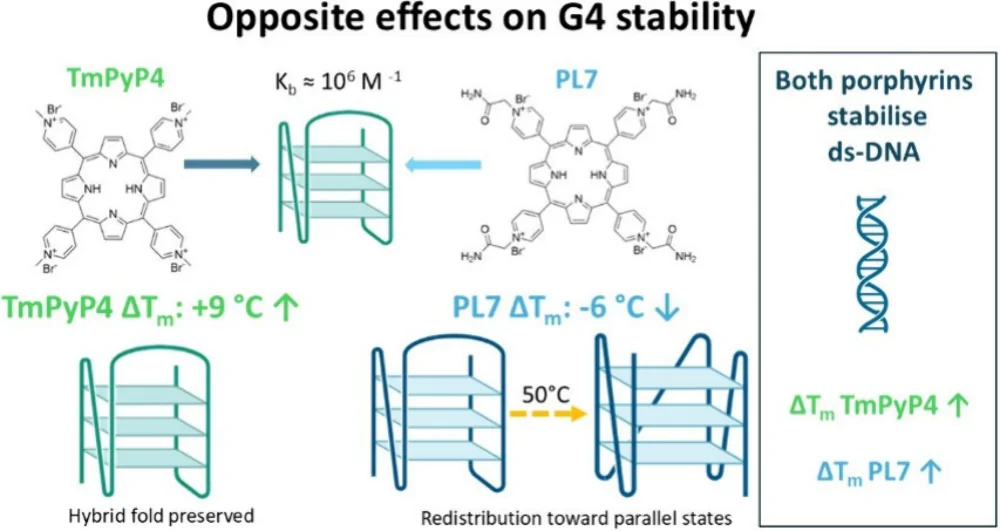

The interaction of small molecules with G-quadruplex (G4) DNA is commonly interpreted through the lens of binding affinity, which is often taken as a proxy for structural stabilization and biological relevance.^1,2^ G4s are non-canonical nucleic acid structures formed by stacked guanine tetrads and stabilized by monovalent cations.^3–7^ Because of their involvement in gene regulation and genome stability, considerable efforts have been devoted to the discovery of ligands capable of selectively recognizing and stabilizing G4 structures.^3–6,8–12^ Among G4 ligands, cationic porphyrins represent one of the most extensively investigated families owing to their strong electrostatic and π-stacking interactions with quadruplex DNA.^13–16^ However, increasing evidence suggests that binding and stabilization may not necessarily be coupled. Zamiri et al. reported that ligand binding may induce distortion or refolding,^17^ while Joshi and co-workers demonstrated that TMPyP3 and TMPyP4 porphyrins strongly bind a parallel G4 within the human *MRP1* gene while simultaneously destabilizing its folded structure.^18,19^ A recent review further highlighted ligand-induced G4 destabilization as an emerging outcome of ligand binding.^20^

To this end, we selected TMPyP4, a well-established G4-binding porphyrin, as a benchmark ligand and compared it with the recently synthesized PL7, a structurally related tetracationic porphyrin (Fig.[Fig fig1]).^21^ Motivated by these observations, we sought to determine whether the apparent disconnect between affinity and stabilization extends beyond the previously reported MRP1/TMPyP3–TMPyP4 system. Using UV–Vis binding studies, thermal denaturation, circular dichroism spectroscopy, and molecular docking, we investigated their interactions with duplex DNA and the human telomeric Tel22 G4. The CD spectrum of G4, measured in the presence of potassium phosphate buffer (Figure S1, Supporting Information file), shows a positive band at 218 nm, a negative band at 238 nm and an intense positive band at 292 nm indicative of a K^+^ driven hybrid Tel22 G4 structure,^22,23^ in agreement with previous CDs reported in the literature.^24–26^ Porphyrin binding to duplex and G4 DNA was assessed by UV–Vis titrations in potassium phosphate buffer (Figure 2 and Figure S2). The resulting spectrophotometric curves were used to determine the corresponding binding constants (*K*_b_) using the Benesi–Hildebrand method (Figure S3–S4, Table S1).^27^ Both porphyrins exhibited strong affinity for both DNA forms, with binding constants in the 10^4^–10^6^ M^–1^ range and a clear preference for G4 over ds-DNA (Table 1). The magnitude of the binding constants and the observed hypochromic and bathochromic shifts are consistent with strong π–π stacking and electrostatic interactions at the DNA interface, indicating effective complex formation in both systems. Van’t Hoff analysis of the temperature-dependent binding constants yielded the thermodynamic parameters ΔH and ΔS from the slope and intercept of the corresponding linear plots (Figure S5). These parameters provide insight into the molecular forces governing ligand association and reveal whether binding is primarily driven by enthalpic or entropic contributions.^28,29^

**Figure 1:**
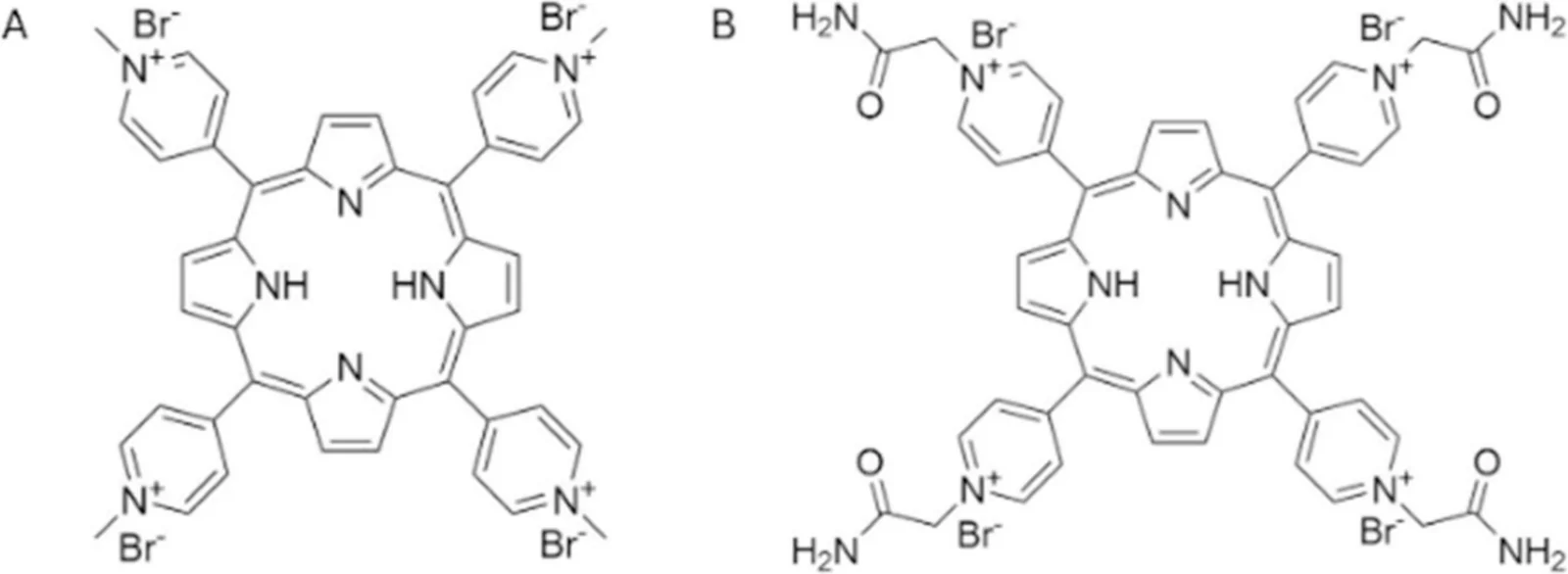
Molecular structures of TMPyP4 (A), used as a benchmark G4-binding porphyrin, and PL7 (B), a structurally related tetracationic porphyrin investigated in this work.

**Table 1:** Thermodynamic Parameters of Porphyrin/ds-DNA and /G4 Interactions in 25 mM Potassium Phosphate Buffer at pH 7.4. Binding constants (*K*_b_), and Corresponding Δ*G*° Values, Were Obtained at 298 K^a^

sample	*K*_b_ (M^–1^)	Δ*H* (kJ mol^–1^)	Δ*S* (J mol^–1^ K^–1^)	Δ*G* (kJ mol^–1^)
**ds-DNA/TMPyP4**	(3.6 ± 0.7) × 10^4^	+7 ± 2	+111 ± 5	–25.9 ± 0.2
**ds-DNA/PL7**	(4 ± 1) × 10^4^	+46 ± 23	+245 ± 78	–26.2 ± 0.6
**G4/TMPyP4**	(1.6 ± 0.2) × 10^6^	–17 ± 11	+57 ± 38	–35.3 ± 0.2
**G4/PL7**	(2.5 ± 0.4) × 10^6^	+15 ± 12	+170 ± 43	–36.4 ± 0.2

a Δ*H* and Δ*S* values were obtained from van’t Hoff analysis of *K*_b_ measured at different temperatures.^30^

**Figure 2:**
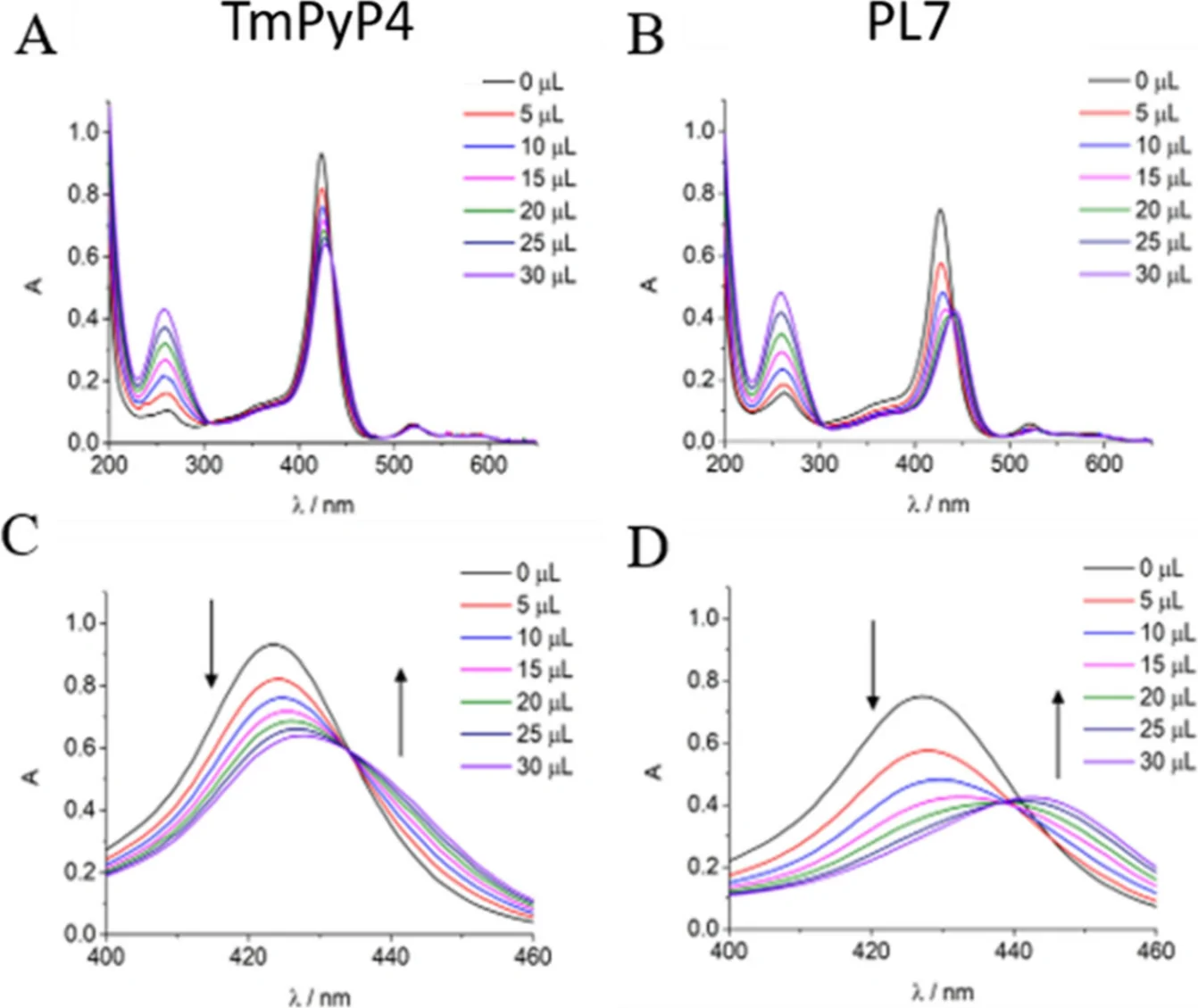
UV-Vis spectra at 25 °C of 5 μM porphyrins in potassium phosphate buffer 25 mM (A–B) and magnified views (C–D) upon addition of 0.68 mg mL^–1^ G4.

Although TMPyP4 and PL7 exhibit comparable binding affinities toward G4 DNA, their thermodynamic signatures differ substantially (Table 1). TMPyP4 binding is characterized by a favorable enthalpic contribution (Δ*H* = −17 kJ mol^–1^) accompanied by a moderate entropic gain, whereas PL7 binding displays an unfavorable enthalpy (Δ*H* = +15 kJ mol^–1^) compensated by a markedly larger entropy increase (Δ*S* = +170 J mol^–1^ K^–1^). These results indicate that similar overall free energies of binding can arise from distinct molecular driving forces. In particular, the strongly entropy-driven association observed for PL7 may reflect a larger contribution from solvent release, counterion displacement, and ligand-induced conformational rearrangements within the quadruplex structure.

To determine whether these distinct binding thermodynamics translate into different structural outcomes, thermal denaturation experiments were performed. Thermal denaturation measurements, shown in Figure 3, reveal an opposite trend of the melting profiles due to the different spectral responses of ds-DNA and G4. UV absorption arises mainly from π → π* electronic transitions of the nucleobases, whose intensity is strongly affected by base stacking. In ds-DNA, base stacking within the double helix reduces the absorbance at 260 nm. As the temperature increases, thermal denaturation disrupts hydrogen bonding and π–π stacking, producing a characteristic hyperchromic increase in absorbance.^31^ In G4 DNA, the stacking of G-quartets also contributes significantly to the spectroscopic response; however, unfolding is more effectively monitored at 295 nm, where disruption of Hoogsteen hydrogen bonding and quartet stacking results in a progressive decrease in absorbance.^32,33^ The melting temperature (*T*_m_) was determined as the midpoint of the spectroscopic unfolding transition (Figure S6) and used to compare the relative stability of DNA in the absence and presence of the porphyrins. Accordingly, positive and negative shifts in *T*_m_ relative to ds-DNA or G4 indicate thermal stabilization and destabilization, respectively (Table 2).

**Figure 3:**
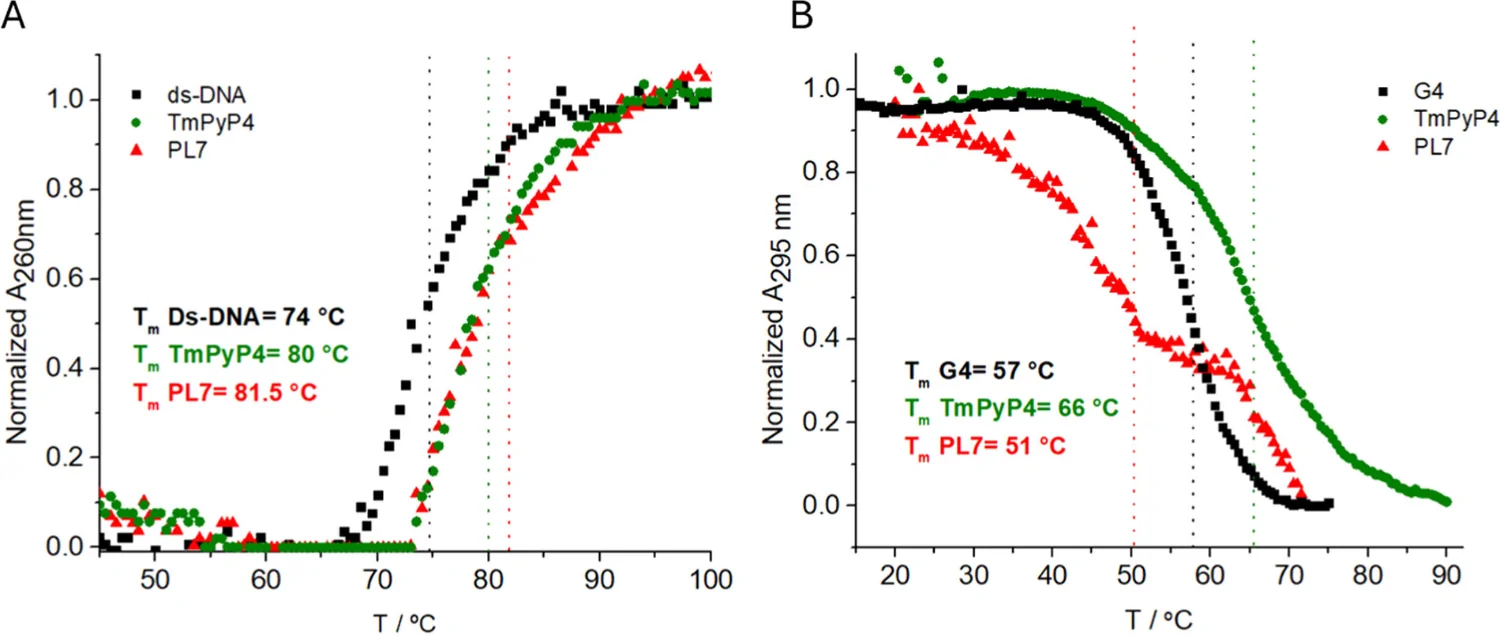
Representative melting curves of free ds-DNA and Tel22 G4 and their complexes with porphyrins. (A) 1 mg mL^–1^ ds-DNA and (B) 0.68 mg mL^–1^ G4 in 25 mM potassium phosphate buffer at pH 7.4.

**Table 2:** Melting Temperature (*T*_m_) Values in 25 mM Potassium Phosphate Buffer at pH 7.4^a^

sample	control	TMPyP4	PL7	TMPyP4	PL7
	*T*_m_ (°C)	*T*_m_ (°C)	*T*_m_ (°C)	Δ*T*_m_	Δ*T*_m_
**ds-DNA**	73.7 ± 0.4	75.7 ± 0.6	76.5 ± 0.8	2 ±1	3 ± 1
**G4**	57.1 ± 0.5	66 ± 1	51 ± 2	9 ± 1	–6 ± 2

a Melting temperature difference (Δ*T*_m_) with respect to ds-DNA (260 nm) and G4 (295 nm) due to the interaction with porphyrins (Δ*T*_m_ = *T*_m_ DNA/P - *T*_m_ DNA). *T*_m_ values were determined graphically for each replicate and are reported as mean ± SD based on at least three independent measurements.

Thermal denaturation experiments reveal that both TMPyP4 and PL7 stabilize ds-DNA, as shown by positive Δ*T*_m_ values in potassium phosphate buffer (+2.0 °C and +2.8 °C, respectively). While TMPyP4 increases the G4 melting temperature (+9.0 °C), indicating stabilization of the folded structure, PL7 induces a significant destabilization (Δ*T*_m_ = −6.0 °C). This difference is particularly noticeable given the similar binding affinity of the two ligands toward G4. Despite differing only in the nature of the peripheral pyridinium substituents, the two porphyrins produce opposite effects on G4 thermal stability.

To better understand the origin of the opposite thermal responses induced by TMPyP4 and PL7, circular dichroism (CD) spectroscopy was used to monitor the evolution of G4 topology upon ligand binding and heating (Figure 4). In the absence of ligand, the CD spectrum recorded at 30 °C displays a dominant positive band near 290 nm, a secondary contribution in the 260–275 nm region, and a negative band around 240 nm, consistent with a predominantly hybrid telomeric G4 topology in potassium-phosphate.^25,34,35^ Upon heating, the overall spectral profile is initially retained but progressively loses intensity while preserving the characteristic features of the hybrid fold (Figure 4A). At elevated temperatures, the marked attenuation of the 290 nm band indicates progressive disruption of the quadruplex structure, including the loss of ordered G-quartet stacking and thermal unfolding. In the presence of TMPyP4, however, the CD spectrum at 30 °C retains the characteristic features of the hybrid telomeric G4 topology (Figure 4B). Upon heating, the overall spectral profile remains largely unchanged apart from a gradual decrease in intensity, indicating preservation of the native quadruplex architecture over a broad temperature range. This behavior is consistent with the positive Δ*T*_m_ observed in thermal denaturation experiments. The observed shifts reflect the relative stabilization of the conformational states involved in the thermal transition and cannot be interpreted solely in terms of ligand affinity for the folded G4. Binding to partially unfolded or alternative conformations may also contribute to the apparent thermal response. Since state-specific binding constants for these conformations were not determined here, such contributions cannot be excluded.

**Figure 4:**
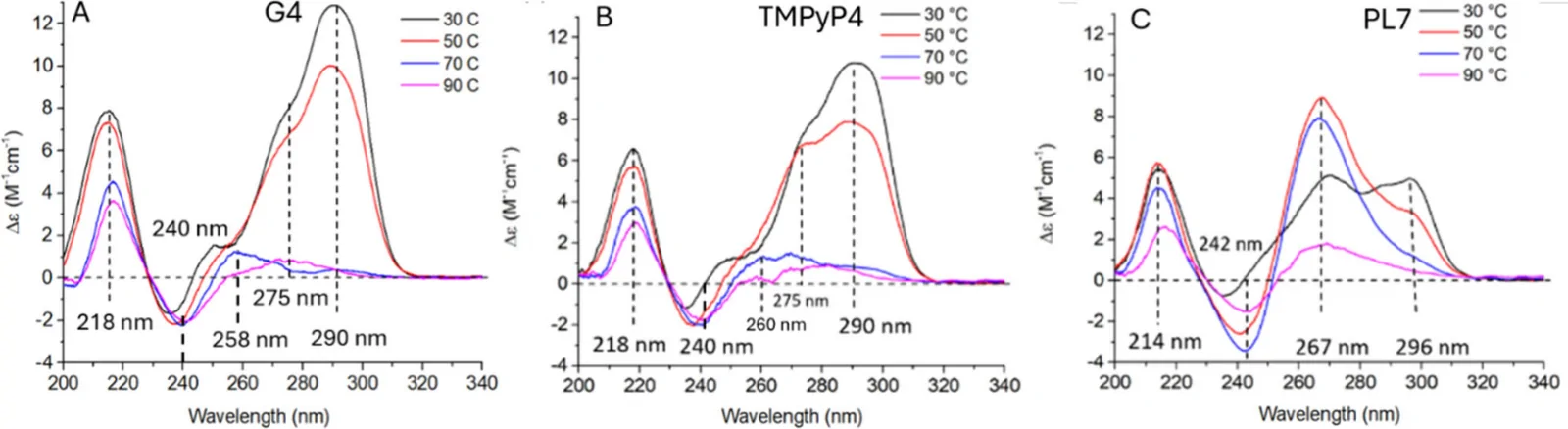
CD spectra of G4 0.68 mg mL^–1^ (A) with TMPyP4 (B) or PL7 (C) 12 μM in a range of temperatures assembled in potassium phosphate buffer 25 mM at pH 7.4.

A markedly different behavior is observed for PL7 (Figure 4C). At 30 °C, the CD spectrum remains characteristic of a folded G4 structure. However, upon heating, the contribution in the 260–270 nm region becomes progressively more pronounced, whereas the intensity of the band near 290 nm decreases. This change of the CD signal is consistent with a greater contribution from parallel G4 conformations rather than with a simple loss of quadruplex folding.^21,35–38^ These results suggest that PL7 modifies the equilibrium among various conformational states. The complex temperature dependence may reflect either a gradual redistribution among coexisting conformational states or one or more discrete conformational transitions. Accordingly, the apparent decrease in *T*_m_ should not be interpreted solely as global G4 unfolding. Preferential PL7 binding to specific conformational states, including states populated at higher temperature, may also contribute to the observed thermal response. The present CD data do not allow state-specific binding affinities to be determined. To investigate the structural basis of the contrasting thermal and conformational effects induced by TMPyP4 and PL7, molecular docking studies were performed. Special emphasis was placed on the unusual behavior of PL7, which displays high G4 affinity while reducing quadruplex thermal stability. To account for the structural polymorphism of human telomeric G4s in K^+^ solution, calculations were performed on both the experimentally determined hybrid-1 (PDB ID: 2HY9; Figure 5) and hybrid-2 (PDB ID: 2JPZ; Figure S7) topologies, which have been reported to coexist in equilibrium in K^+^ solution.^25,34^ The interactions between the studied porphyrins and the Tel22 G4 may arise from π–π stacking between the porphyrin macrocycle and exposed nucleobases, either in the tetrads or in the loops; electrostatic attraction between the pyridinium groups and the negatively charged backbone; and hydrogen bonding between the peripheral groups of the ligand and loop or groove residues. The relative contribution of these interactions is expected to depend on the binding geometry. Consistent with this view, the docking calculations indicate the accessibility of multiple binding modes involving stacking, groove-associated, and hydrogen-bonding interactions. In the hybrid-1 structure (2HY9), terminal G-tetrads are partially shielded by loop nucleobases, limiting the accessibility of ideal end-stacking geometries. Accordingly, docking predicts several alternative PL7 binding modes involving loop-associated and groove-associated regions in addition to external tetrad binding (Fig. 5A–C). Similar results were obtained for the hybrid-2 structure (2JPZ, Figure S7), where both external and lateral binding modes were identified. Overall, the calculations indicate that multiple energetically accessible binding modes are possible in both telomeric G4 topologies. Such binding-mode diversity may contribute to the strongly entropy-driven association of PL7 by enabling interactions with a broader conformational ensemble of telomeric G4 structures. Interactions involving loops and grooves, which are known to influence telomeric G4 topology and dynamics, may play a key role in the shifting of the conformational equilibrium.^14–16,34,36–38^ Although docking calculations cannot uniquely identify the experimentally populated binding mode, the predicted structural diversity provides a plausible framework for interpreting the apparent decoupling between affinity and thermal stabilization.

**Figure 5:**
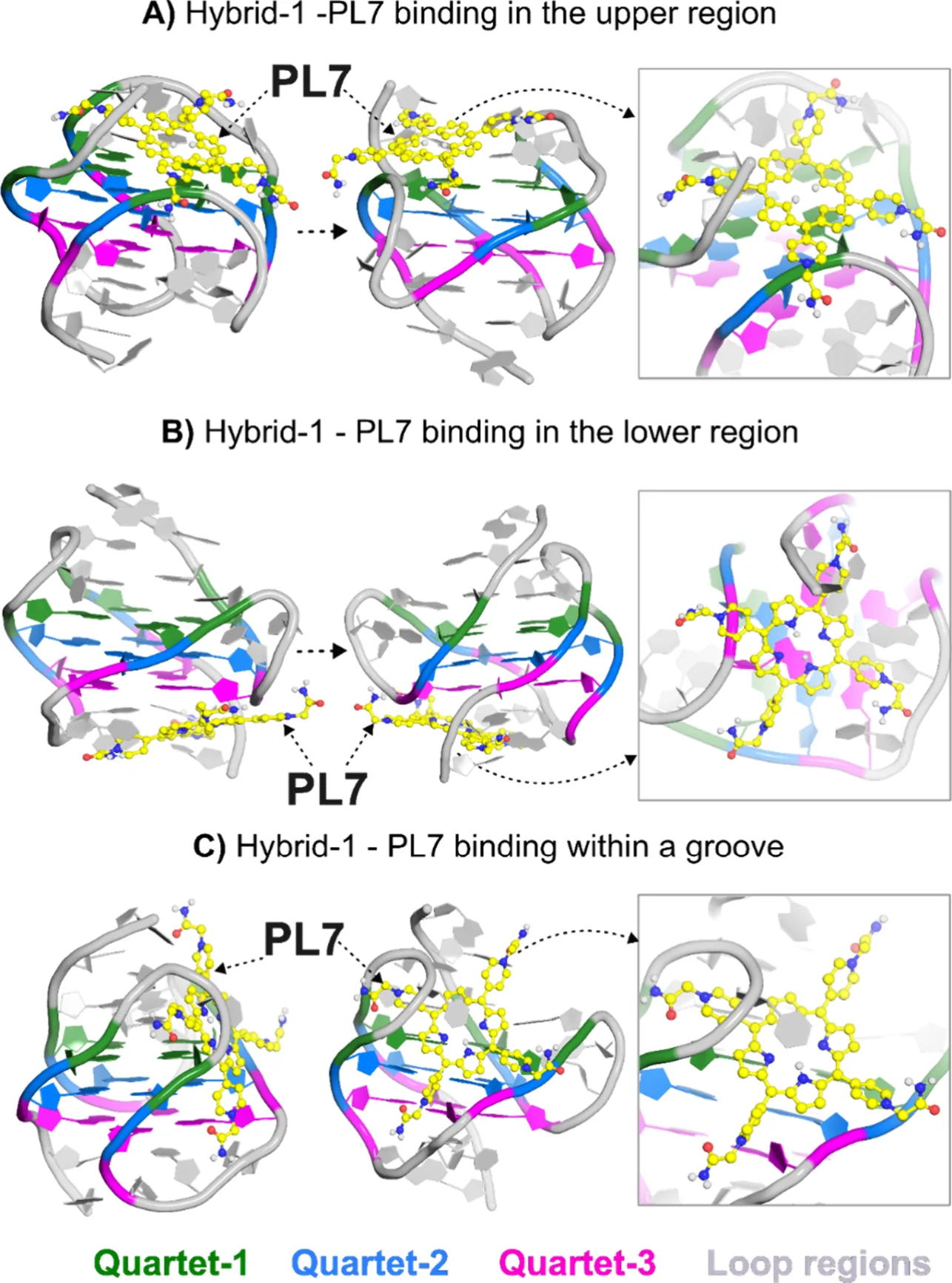
Representative docking poses of PL7 with the hybrid-1 human telomeric G4 (PDB ID: 2HY9). Three docking poses are shown, corresponding to ligand binding in the upper external region (A), the lower external region (B), and within a groove (C) of the G4. For each pose, two views and an enlarged detail of the binding region are provided. The three G-tetrads are coloured green, blue, and magenta, respectively, whereas the loop regions are shown in light grey. PL7 is shown in yellow using a ball-and-stick representation. The coexistence of multiple binding geometries is qualitatively consistent with the entropy-driven binding of PL7 and its ability to redistribute the telomeric G4 conformational ensemble.

Despite comparable *K*_b_ values, TMPyP4 and PL7 exhibit distinct thermodynamic signatures and opposite effects on quadruplex thermal stability, revealing a decoupling between ligand binding and G4 stabilization. While binding affinity reflects the overall free energy of association, thermal stability reports on the persistence of the folded quadruplex topology.^29,40^ Building on previous observations,^18,19^ our results demonstrate that high-affinity ligands can exert markedly different effects on G4 structure and stability. Combined thermodynamic, spectroscopic, and computational analyses suggest that ligand-induced conformational redistribution can destabilize the native quadruplex fold despite favorable binding. Together, these results challenge the assumption that stronger binding implies greater stabilization and redefine affinity and stability as independent determinants of G4-ligand interactions.

## Supplementary Material



## Data Availability

All data are available from the lead contact upon reasonable request.

## Notes

Materials and experimental procedures; CD characterization of G4 folding; UV–Vis titrations of porphyrins with ds-DNA and G4 DNA; determination of binding and thermodynamic parameters; thermal melting experiments and graphical *T*_m_ determination; supplementary figures and tables; molecular docking methodology and representative binding poses (PDF)
