# Supplementary material for: Binding Does Not Mean Stabilizing: High-Affinity Porphyrins Exhibit Opposite Effects on G‑Quadruplex Stability
Source: J Phys Chem Lett. 2026 Sep 14;17(38):10919–25. doi: 10.1021/acs.jpclett.6c02493 (PMC13618040; doi:10.1021/acs.jpclett.6c02493)
Supplement: Supplementary file 1 [file jz6c02493_si_001.pdf]

Binding Does Not Mean Stabilizing: High-Affinity Porphyrins Exhibit Opposite Effects on G-Quadruplex Stability

*Cristina Carucci,[a] Monica Mura,[a] Francesca Cherchi,[a] Giuseppe Satta,[b] Aamir Saeed,[a] Barbara Jachimska,[c] Francesca Mocci,[a] Massimo Carraro,[b] Andrea Salis[a]**

[a]Department of Chemical and Geological Sciences, University of Cagliari & CSGI, Cittadella Universitaria, S.S. 554 bivio Sestu, 09042 Monserrato (CA), Italy.

[b]Department of Chemical, Physical, Mathematical and Natural Sciences, University of Sassari, Via Vienna 2, Sassari, 07100, Italy

[c]Jerzy Haber Institute of Catalysis and Surface Chemistry Polish Academy of Sciences, Krakow, Poland.

EXPERIMENTAL

Chemicals

Monobasic sodium phosphate (99 %), disodium hydrogen phosphate (99 %), monobasic potassium phosphate (99 %), dipotassium hydrogen phosphate (99 %) were purchased from Sigma-Aldrich. Pyrrole was purchased from Sigma-Aldrich and distilled prior to use. 4-pyridinecarboxaldehyde and 2-bromoacetamide were purchased from TCI. Distilled water was purified through a Millipore system (Simplicity 185). Highly polymerized calf thymus ds-DNA was purchased from Sigma-Aldrich, with 6 % w/w sodium content. The lyophilized guanine-rich oligonucleotide having 22 nitrogenous bases (Tel22 5'-AGGGTTAGGGTTAGGGTTAGGG-3'), characteristic sequence of telomeres, was purchased from Merck. TMPyP4 and PL7 were synthesised following a literature procedure.[1] The corresponding structures are reported in Fig. 1.

Preparation of ds-DNA solutions

Phosphate buffers (PB) were prepared by mixing 25 mM acid solution and 25 mM basic solution until they reached pH 7.4. Ds-DNA solutions (1 mg mL^{-1}) were prepared by weighing 4 mg of dried DNA and adding 4 mL of PB. The purity of the duplex DNA stock solution was assessed by UV–Vis spectroscopy before use. DNA concentration and purity were evaluated from the absorbance at 260 nm and from the A_{260}/A_{230} and A_{260}/A_{280} ratios, which were required to be ≥ 2.0 and ≥ 1.8, respectively. The vial was put in slow rotation at 25 °C until a perfectly homogeneous and transparent solution was obtained. CT-DNA solutions were stored at 25 °C and slowly rotated for 24 h before any use. Molar concentration was determined through UV-Vis spectroscopic measurements ($\epsilon = 6600$ M^{-1} cm^{-1}) and appropriate dilutions were made before experiments.

Assembly of G4

Tel22 100 μM solutions were prepared by dissolving the solid oligomers (186 nmol, 1.3 mg) in 1.860 mL of K^+ PB at room temperature for 20 minutes. The solution was then transferred into a quartz cuvette with a PTFE sealing cap and heated gradually at 90 °C using a Peltier-effect temperature-control module. Temperature was maintained for 10 minutes, then slowly decreased in steps of 1 °C

/ min to 25 °C. The G4 solution was divided into 400 μL aliquots inside 1 mL glass vials and frozen to be thawed when necessary. Before using the aliquot, it was left at 25°C in rotation overnight. The assembly of G4 structures was assessed by circular dichroism analysis as described below.

Circular Dichroism (CD) measurements

G4 samples were prepared as described above. CD spectra were recorded for 100 μM G4 in 25 mM PB at pH 7.4, both in the absence and in the presence of 12 μM porphyrins. For temperature dependent CD measurements, G4/porphyrin samples were equilibrated directly in the cuvette at each selected temperature for at least 3 min before CD spectra acquisition. CD spectra were recorded using a Jasco J-1500 spectropolarimeter equipped with a Peltier-type temperature control system (model PM-539). Far-UV CD spectra were collected in 10 mm path-length quartz cuvettes (Hellma Analytics, Southend-on-Sea, UK) at 25 °C in the 200–350 nm range, at a scanning speed of 100 nm/min, a response time of 1s, a bandwidth of 2 nm, and a data pitch of 0.1 nm. The spectra were signal-averaged over 5 scans and baseline-corrected by subtracting a buffer spectrum.

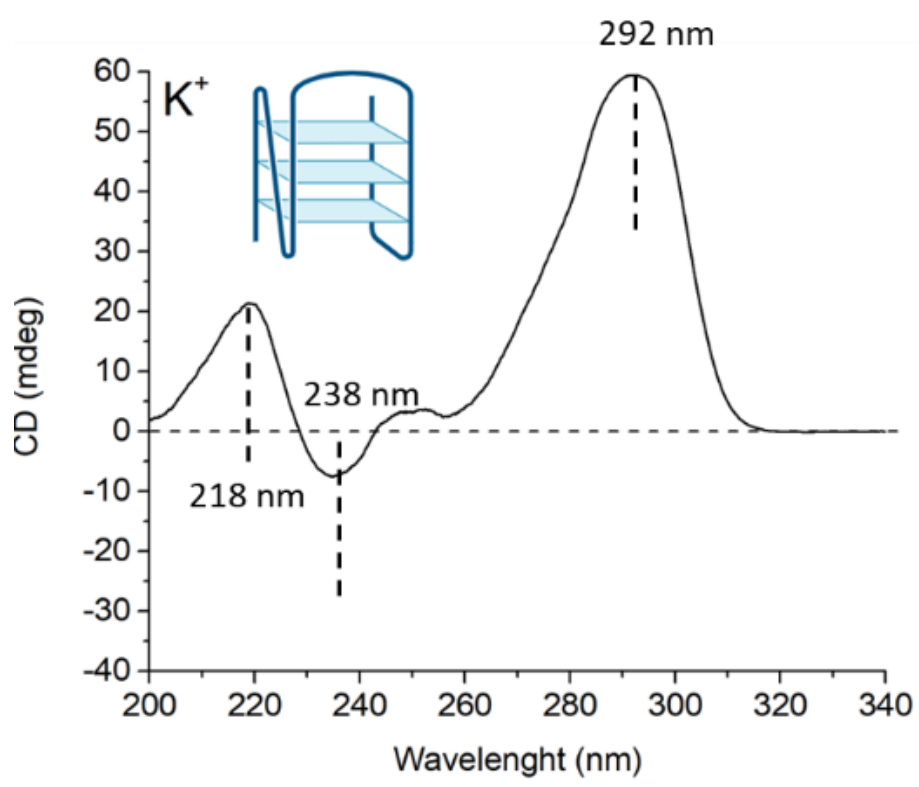

Figure S1. CD spectra of G4 0.68 mg mL^{-1} assembled in potassium phosphate buffer 25 mM at pH 7.4.

UV-Vis titrations

The binding constant (K_b) of porphyrins to double strand DNA (ds-DNA) and G-quadruplex DNA (G4) was determined by means of spectrophotometric titrations. Porphyrin solutions 5 μM in PB (Na^+ or K^+) 25 mM at pH 7.4 were analysed in the UV-Vis region (200-800 nm) after subsequent aliquots of DNA were added. The Benesi-Hildebrand method was applied to experimental data to obtain K_b.[2]

$$\frac{A_0}{A - A_0} = \frac{\varepsilon_P}{\varepsilon_{DNA:P} - \varepsilon_p} + \frac{\varepsilon_P}{\varepsilon_{DNA:P} - \varepsilon_p} \cdot \frac{1}{K_b \cdot [DNA]} \tag{S1}$$

Where: A_0 and A are the absorbance of free porphyrin and of the porphyrin–DNA solution; ε_p and $\varepsilon_{DNA:P}$ represent the molar extinction coefficients of the porphyrin and DNA/porphyrin complex, respectively. K_b is the binding constant and $[DNA]$ is the molar DNA concentration.

Titrations were carried out at different temperatures at 15, 20, 25, and 30 °C to obtain thermodynamic parameters, ΔH and ΔS through van't Hoff plot.[3]

$$lnK_b = -\frac{\Delta H}{RT} + \frac{\Delta S}{R} \tag{S2}$$

Where R is the gas constant. The intercept and slope of the van't Hoff plot of lnK_b against 1/T (expressed in K) were used to calculate the thermodynamic parameters ΔS (entropy) and ΔH (enthalpy) values. The free energy at each temperature was calculated as:

$$\Delta G° = -RTlnK_b \tag{S3}$$

Table S1. Binding constants (K_b) for TMPyP4 and PL7 with ds-DNA and G4 DNA at different temperatures.

DNA	Ligand	K_b (M^{-1}) 15 °C	K_b (M^{-1}) 20 °C	K_b (M^{-1}) 25 °C	K_b (M^{-1}) 30 °C
**ds-DNA**	**TMPyP4**	$(3.3 \pm 0.6) \times 10^4$	$(3.2 \pm 0.5) \times 10^4$	$(3.6 \pm 0.7) \times 10^4$	$(3.9 \pm 0.5) \times 10^4$
**ds-DNA**	**PL7**	$(2.5 \pm 0.5) \times 10^4$	$(3.6 \pm 0.1) \times 10^4$	$(4 \pm 1) \times 10^4$	$(4.5 \pm 0.1) \times 10^4$
**G4**	**TMPyP4**	$(1.8 \pm 0.1) \times 10^6$	$(1.8 \pm 0.9) \times 10^6$	$(1.6 \pm 0.2) \times 10^6$	$(2.1 \pm 0.3) \times 10^6$
**G4**	**PL7**	$(1.5 \pm 0.2) \times 10^6$	$(1.4 \pm 0.8) \times 10^6$	$(2.5 \pm 0.4) \times 10^6$	$(1.5 \pm 0.4) \times 10^6$

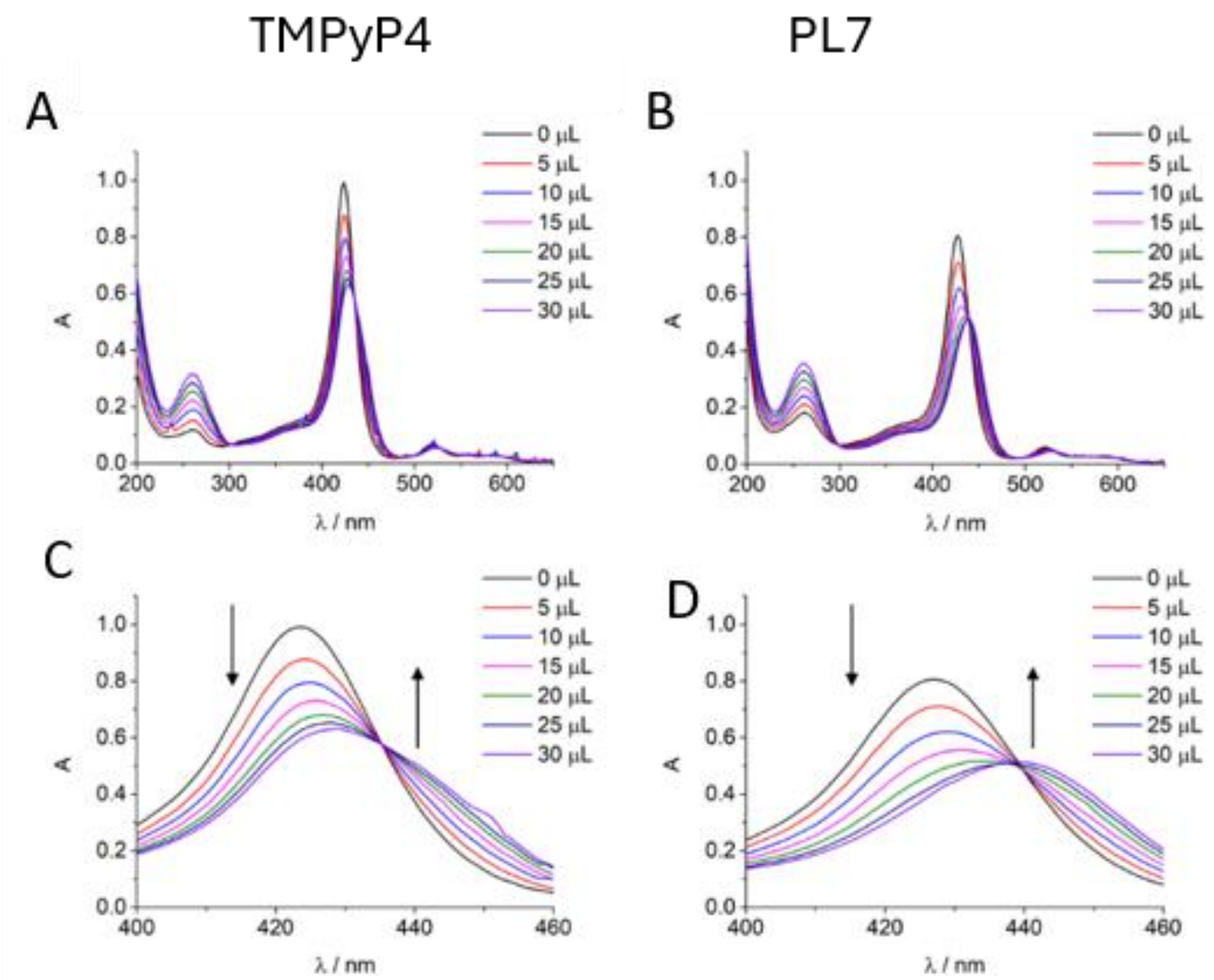

Figure S2. UV-Vis variation spectra at 25 °C of 5 µM porphyrins in potassium phosphate buffer 25 mM (A-B) and zoom (C-D) by adding 1 mg mL^{-1} ds-DNA. Arrows indicate the bathochromic shift.

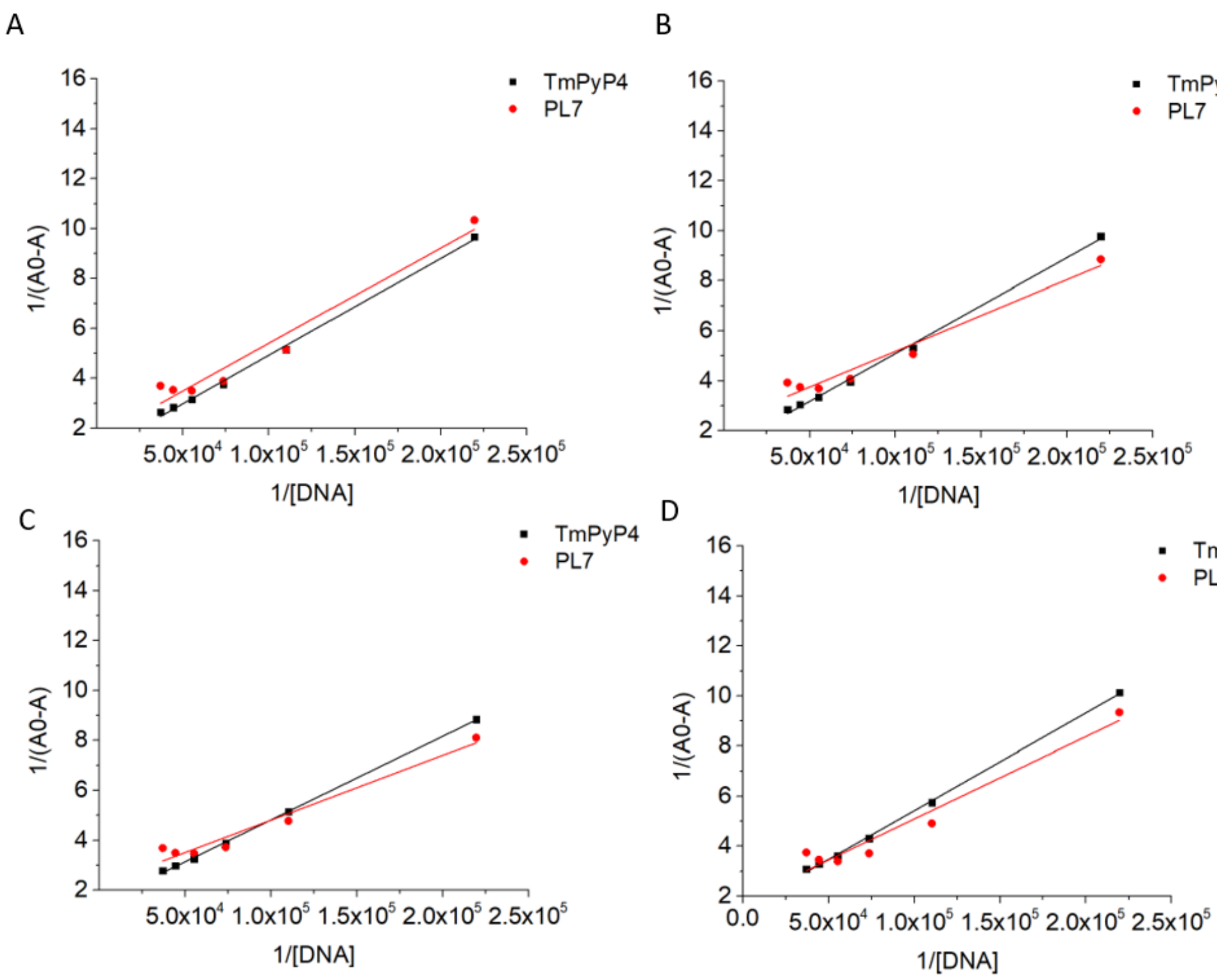

Figure S3. Benesi-Hildebrand plot relative to titrations porphyrins/ds-DNA in 25 mM potassium phosphate buffer varying the temperatures 15°C, 20°C, 25°C, 30°C (A, B, C, D).

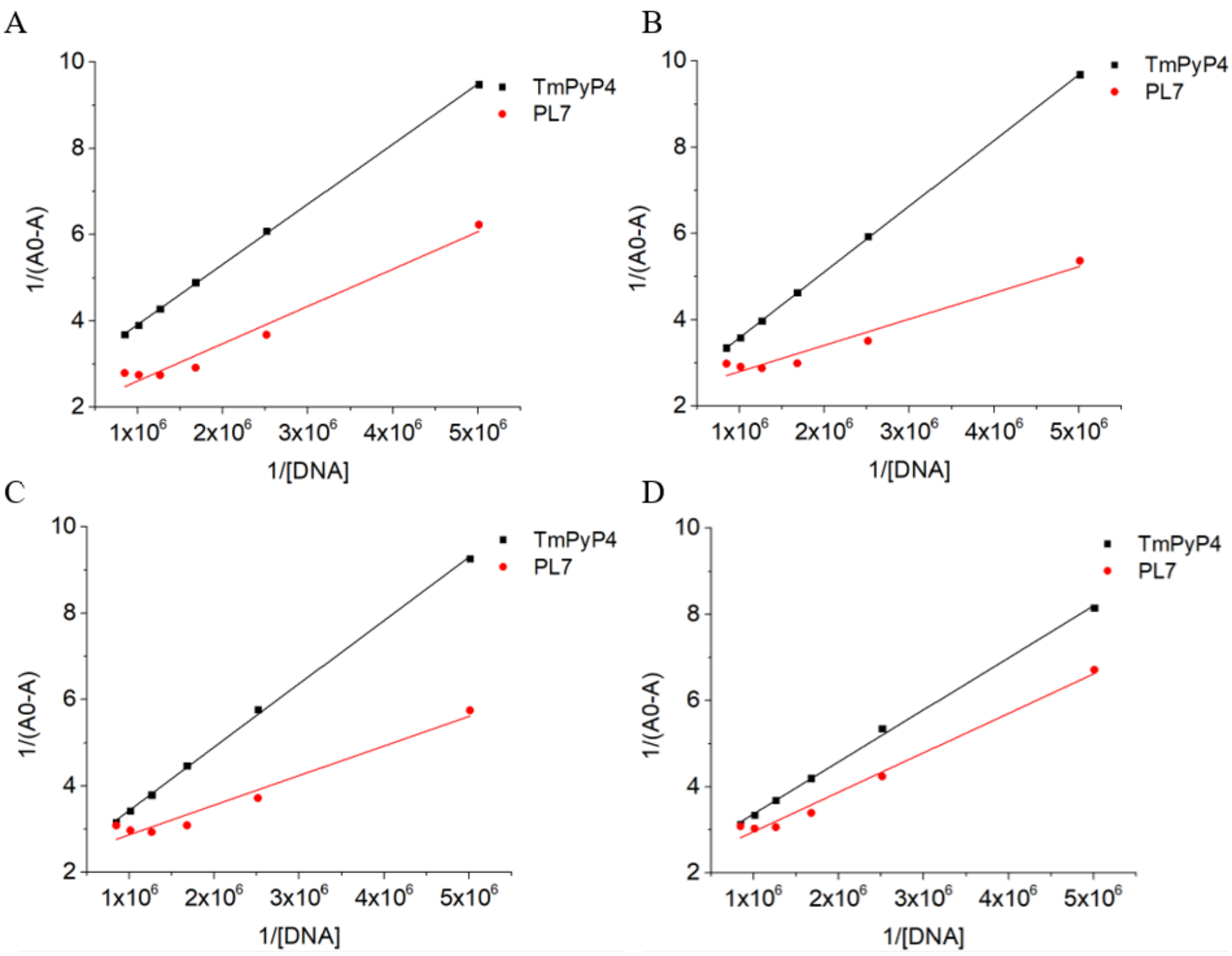

Figure S4. Benesi-Hildebrand plot relative to titrations porphyrins/G4 in 25 mM potassium phosphate buffer varying the temperatures 15°C, 20°C, 25°C, 30°C (A, B, C, D).

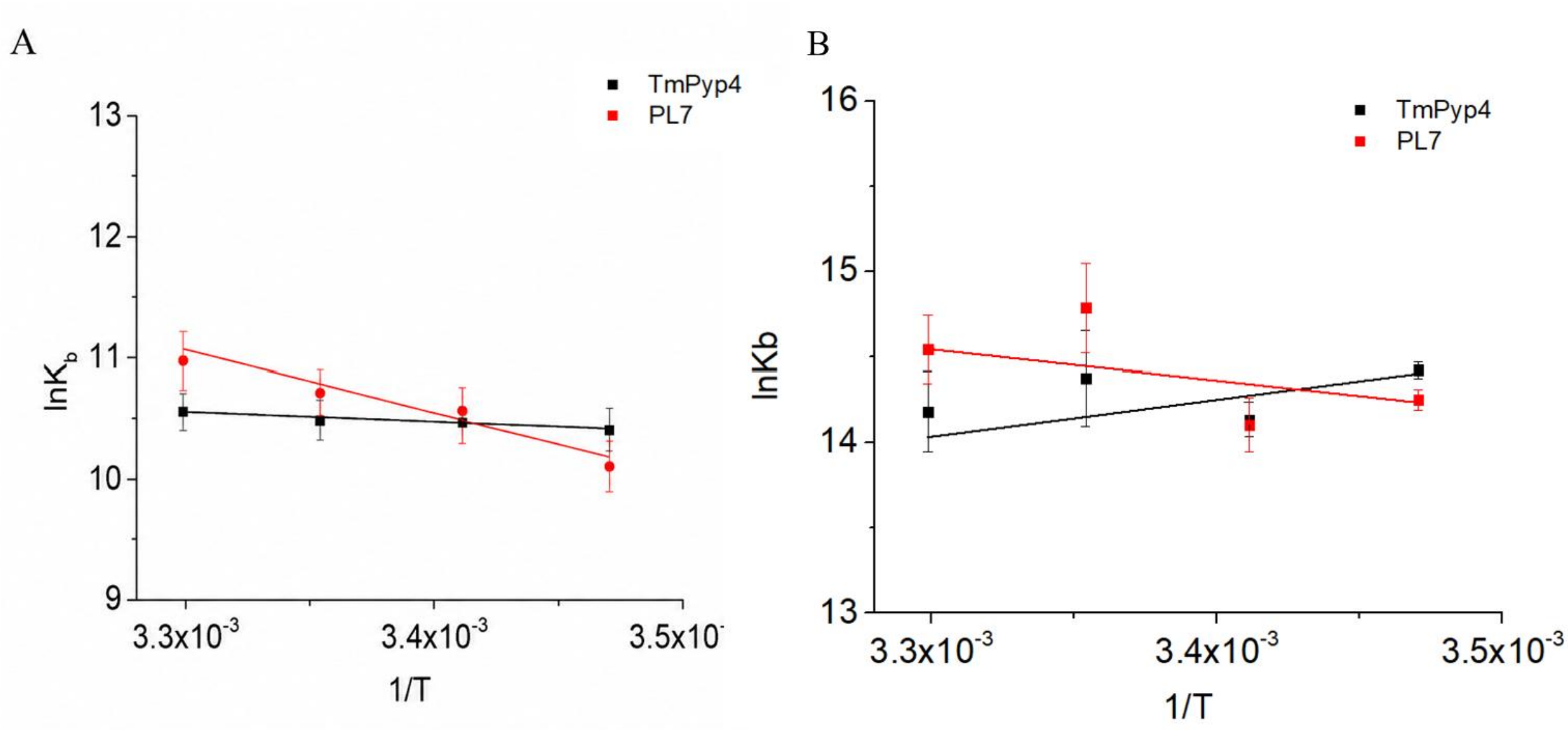

Figure S5. van't Hoff plot with porphyrins /ds-DNA (A) and with porphyrins/G4 (B) in 25 mM PB-K.

Thermal melting curves

Ds-DNA and G4 melting experiments were carried out at DNA concentrations of 1 mg mL^{-1} and 0.68 mg mL^{-1} G4 respectively together with porphyrins 12 µM in PB (Na^+ or K^+) 25 mM at pH 7.4. Melting

temperature (T_m) was determined by UV-Vis thermal denaturation experiments, increasing the temperature using an Agilent Peltier accessory and every 0.5 °C measuring the change in absorbance at 260 nm for ds-DNA and 295 nm for G4. DNA/P ratio was optimized to observe the melting curve at the temperatures reachable with experimental conditions and observe the effect of interactions.

Melting temperature (T_m) determination

The melting temperature (T_m) was obtained by graphical analysis of the UV–Vis melting curves. Briefly, the pre-transition and post-transition regions of each curve were fitted with linear functions, corresponding to the lower and upper baselines, respectively. A median line was calculated as the point-by-point average between these two baselines. The T_m was then determined as the temperature at which the experimental melting profile intersected the median line in the hyperchromic transition region for ds-DNA and the hypochromic transition region for G4. G4 thermal denaturation was monitored by UV–Vis spectroscopy at 295 nm, a wavelength particularly sensitive to changes in G-quartet stacking. The melting temperature Tm was determined using a graphical baseline method. For each experimental melting profile, the initial pre-transition region, corresponding predominantly to the folded G4 state, and the final post-transition region, were independently fitted with linear functions. A median line was then calculated between the two temperature-dependent baselines by averaging their respective slopes and intercepts. The intersection of the experimental melting profile with this median line was taken as the graphical estimate of Tm, corresponding to the midpoint of the thermal transition. This approach accounts for the temperature dependence of the absorbance of both the folded and unfolded states and is therefore more appropriate than determining Tm from a simple midpoint between fixed absorbance values. Independent replicate measurements were analysed separately, and the reported T_m values are expressed as mean ± standard deviation. Ligand-induced changes in thermal stability were calculated as $\Delta T_m = T_m$ DNA-ligand - Tm DNA.

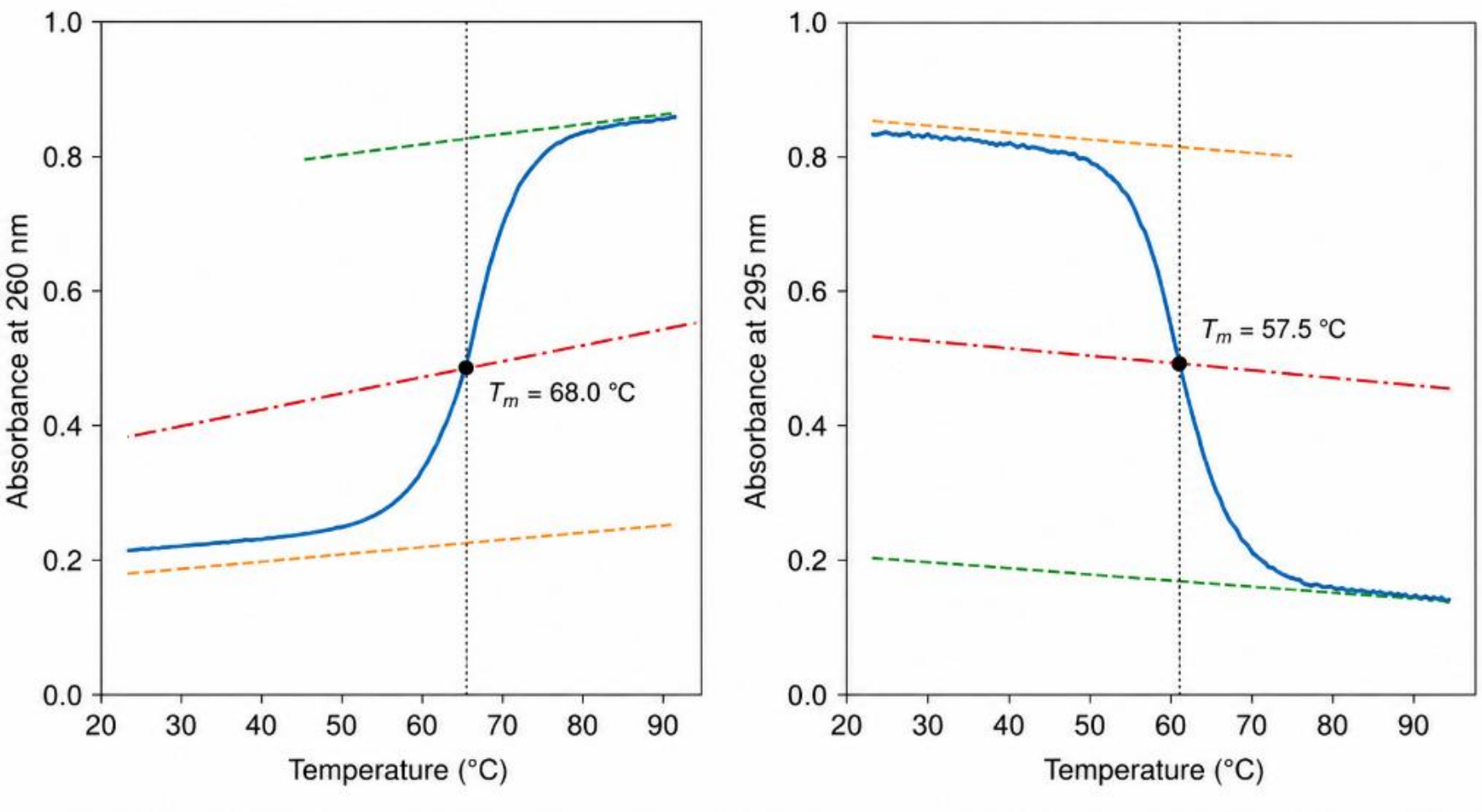

Figure S6. Graphical determination of T_m for (A) duplex DNA at 260 nm and (B) G4 at 295 nm.

Docking calculations

Docking calculations were performed using AutoDock 4.2. The docking search was carried out over the entire G-quadruplex structure, without restricting the ligand to predefined binding sites. AutoDock 4.2 employs a semi-empirical free-energy scoring function that combines van der Waals, hydrogen-bonding, electrostatic, desolvation, and torsional entropy terms. The receptor was treated as rigid, whereas the ligand was treated as fully flexible.

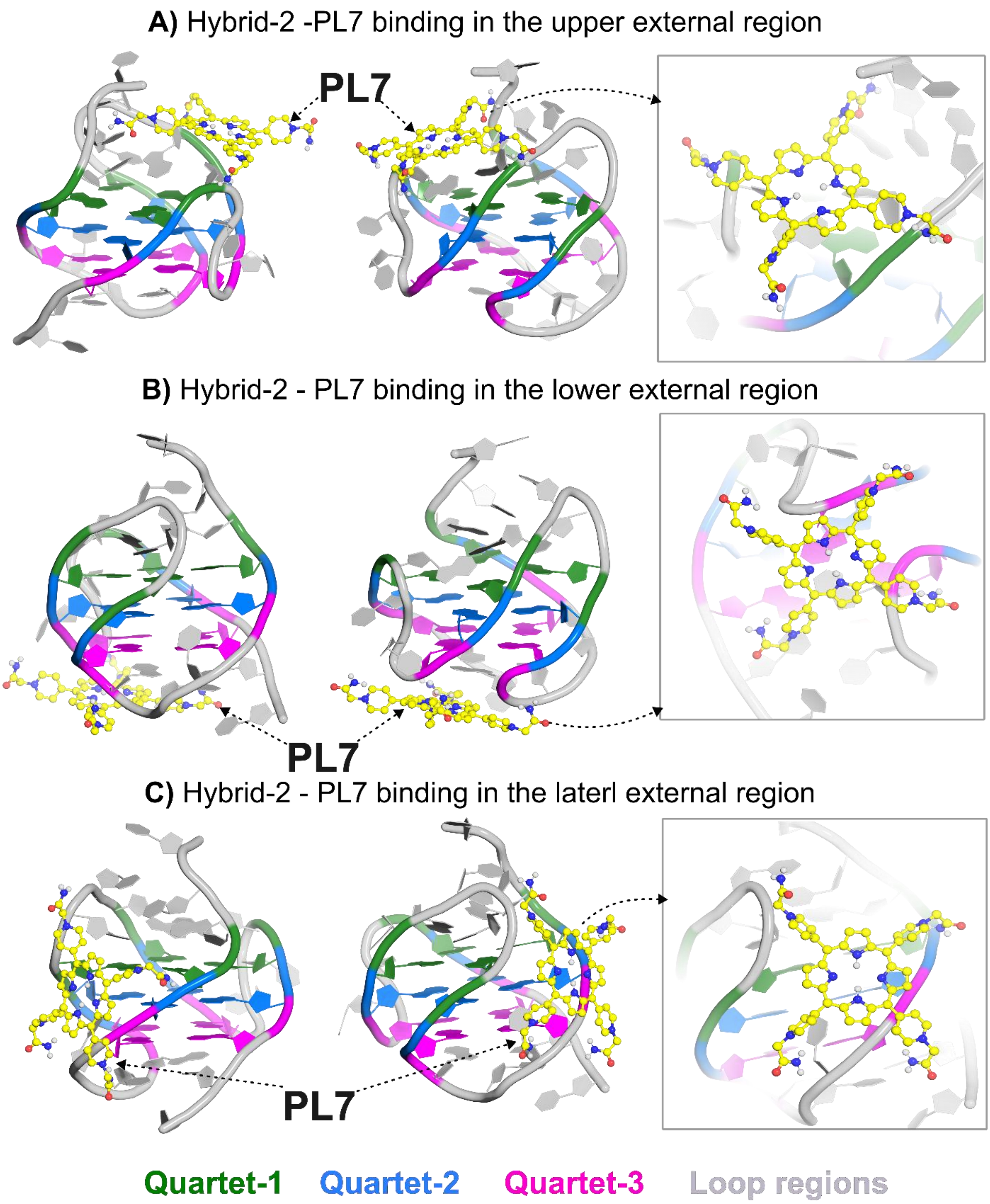

Figure S7. Representative docking poses of PL7 with the hybrid-2 human telomeric G4 (PDB ID: 2JPZ). Three docking poses are shown, corresponding to ligand binding in the upper external region (A), the lower external region (B), and along the lateral surface of the G4 (C). For each pose, two views and an enlarged detail of the binding region are provided. The three G-tetrads are coloured green, blue, and magenta, respectively, whereas the loop regions are shown in light grey. PL7 is shown in yellow using a ball-and-stick representation.

References

1 G. Satta, M. Trajkovski, A. Cantara, M. Mura, C. Meloni, G. Olla, M. Dobrovolná, L.

Pisano, S. Gaspa, A. Salis, L. De Luca, F. Mocci, V. Brazda, J. Plavec and M. Carraro, *Chem. – A Eur. J.*, DOI:10.1002/chem.202402600.

2 H. A. Benesi and J. H. Hildebrand, *J. Am. Chem. Soc.*, 1949, **71**, 2703–2707.

3 R. M. Kenney, K. E. Buxton and S. Glazier, *Biophys. Chem.*, 2016, **216**, 9–18.
